# Post-pandemic era: global trends, benefits, and barriers in integrating artificial intelligence into public health education

**DOI:** 10.3389/fpubh.2025.1648970

**Published:** 2025-10-13

**Authors:** Adewunmi Akingbola, Abiodun Adegbesan, Samuel Tundealao, Akpevwe Emmanuella Benson, Abdulfatai Olumide Makinde, Mayowa Shekoni, Khalid Animashaun, Tosin Fakiyesi

**Affiliations:** ^1^Old Schools Trinity Lane, University of Cambridge, Cambridge, United Kingdom; ^2^Department of Global Health, African Cancer Institute, Stellenbosch University, Cape Town, South Africa; ^3^Department of Biostatistics and Epidemiology, Rutgers School of Public Health, Piscataway, NJ, United States; ^4^Faculty of Pharmacy, University of Benin, Benin, Nigeria; ^5^Faculty of Clinical Sciences, Lagos State University College of Medicine, Ikeja, Nigeria; ^6^Department of Community Health and Primary Healthcare, Lagos State University College of Medicine, Ikeja, Nigeria; ^7^Faculty of Clinical Sciences, University of Ibadan, Ibadan, Nigeria

**Keywords:** COVID-19, public health response, Africa, pandemic preparedness, health systems, vaccine equity

## Abstract

The COVID-19 pandemic posed an unprecedented challenge to public health systems globally, with African countries exhibiting a wide range of outcomes in terms of preparedness, response, and resilience. This review explores the public health strategies deployed across the African continent during the pandemic, highlighting key successes, identifying critical failures, and synthesizing lessons to inform future health emergency preparedness. Drawing on existing literature, policy documents, and epidemiological data, the study examines the roles of leadership, community engagement, health communication, diagnostic capacity, and vaccine deployment. While several African countries successfully leveraged past epidemic experience, decentralized health systems, and innovative communication strategies, others struggled with misinformation, weak surveillance, and limited critical care infrastructure. The review also discusses the role of international collaboration, local innovation, and donor dependence in shaping response outcomes. Lessons from Africa’s handling of COVID-19 underline the importance of strengthening public health infrastructure, investing in health workforce development, improving health information systems, and ensuring equitable access to vaccines and therapeutics. This paper contributes to the growing discourse on pandemic preparedness and highlights Africa’s potential not only as a site of vulnerability but also of resilience and innovation in global public health.

## 1 Introduction

Five years after the onset of the COVID-19 pandemic, the world continues to grapple with its long-term consequences, particularly in the public health sector, which faced significant disruptions and transformations (1, 2). Wuhan Province in China first reported 27 cases of what was called Novel Pneumonia with unknown cause and origin on 31st of December 2019, a swab of the throat taken on January 7, 2020 was analyzed by the Chinese Center for Disease Control and Prevention (CCDC) and was identified as Severe Acute Respiratory Syndrome Coronavirus 2 (SARS-CoV-2) (3, 4). Coronaviruses are frequent ribonucleic acid viruses and belong to the Coronaviridae family and are largely responsible for respiratory and, at other times, digestive infections in humans (1). COVID-19 spread gradually in the world and has thus, became a global pandemic, triggering a health crisis (of which WHO declared a global pandemic on March 11, 2020) (1, 5, 6). This pandemic affected Public Health systems as it represented a global health emergency that brought about exposure to the weaknesses in the structures of healthcare workforces all over the world (1). The effects came in different dimensions, which include complicating already existing workforce shortages and burnout, overstretching the clinical systems, among others (1, 7).

The pandemic made public health practitioners more vulnerable as the responses to COVID-19 required long hours, emotional fatigue, and an increased risk of exposure, which all contribute to the high burnout rates and even a reduction in the workforce (8). Public health systems across both high- and low-income countries struggled to respond effectively as containment measures were permeable and poorly implemented at such a critical point (1, 8, 9). Five years later, it is still unclear whether health care measures in just any country have been employed to cope with the next outbreak because many public health sectors are still bouncing back from the effects of the COVID-19 pandemic (8, 10). One of the lasting impacts of COVID-19 on healthcare is the emergence of Post-COVID-19 Conditions (PCC), also known as long COVID, which refers to ongoing, relapsing, or newly onset multisystem symptoms that occur during the post-acute phase of SARS-CoV-2 infection (11). These conditions, which can persist for up to three years after the initial infection, have affected millions globally—impacting daily functioning, increasing healthcare costs, reducing quality of life, and placing significant strain on the workforce and public sector (12–15). A full recovery from the pandemic’s effects will require a restructured public health systems, one that is well equipped not just for response, but for proactive preparedness through innovations like AI and digital tools. This includes a remodeled approach built around five essential measures: management, protection, containment via control and suppression of transmission, information, and support (12). The increased need for capacity in the public health sector and workforce redistribution post-COVID brought about the growing call for reform in healthcare that includes a long-term investment in the areas of public health training, education, and development of digital skills (1, 12).

The healthcare sector has long struggled with inefficiencies, owing to the intrinsic complexities of which the actor is well known (16). These issues were magnified following the COVID-19 pandemic, during which the overstretched workforce could not meet patients’ needs (16). For this reason, numerous ideas and innovations have been adopted majorly in the areas of capitalizing on the advancement of technologies, but of all the promising technologies, artificial intelligence (AI) stands tall and has proven to be a key possibility, with widespread embrace across several sectors, including the healthcare system (16–19). AI refers to technologies that allows software and machines to perform varying degrees of tasks by duplicating the ability of cognition and the thought processes of humans (16, 20). At the exponential rate of improvement of AI, it can execute and replace an extensive range of human tasks (16). In addition to that, AI can learn from experience and easily adjust to new inputs and new environments as well. All these sets AI with great potential for the healthcare sector, thereby enabling it to overcome numerous challenges and its inefficiencies (16, 20). Taking into consideration drug and vaccine development, AI could facilitate a faster and more effective development of drugs and vaccines, AI could also make a quicker and more accurate detection of diseases and even more accurate prediction of the trajectory of pandemics (21–24).

In addition to all these, AI could also alleviate routine administrative tasks for healthcare professionals and assist in planning and organizing which includes optimal allocation of hospital resources (17). While AI embodies undeniable potential in healthcare, it is also important to note that understanding of the subject is still limited and this is largely due to the disjoint in the nature of previous research (16). The variation in understanding of the performance and improvement brought about by AI may also affect the acceptance of AI (16). Concluding, a lot of AI studies in healthcare are largely based on literature reviews and mostly simulations rather than empirical data of which have also caused a barrier to the understanding the subject (16).

This review examines global progress, persisting barriers, and emerging benefits of integrating AI into public health education—encompassing both public and private healthcare systems and professionals across clinical, administrative, analytical, and educational roles—with a focus on the African region’s digital health reforms, five years post-COVID-19. The selection of a five-year post-COVID-19 lens provides a globally relevant benchmark for assessing the sustained transformation of public health systems, education, and workforce capacity. This timeframe aligns with major strategic frameworks—such as the WHO’s Global Strategy on Digital Health (2020–2025) and the revised International Health Regulations—both of which call for implementation reviews by 2025 (25). It captures a meaningful period in which initial emergency reforms, including the integration of AI into curricula and workforce development, have had time to mature and show measurable outcomes (26, 27). Additionally, 2025 marks a critical window identified by global health institutions for consolidating pandemic lessons and evaluating digital preparedness, making it a logical and impactful point of reflection for this review (28, 29).

As nations across the world continue to rebuild and develop better structures for their health systems, it is of utmost and urgent importance to include AI literacy within public health plans and curricula, not in the form of coding or system design, but as foundational understanding that enables professionals to interpret outputs, use AI-driven tools effectively, and collaborate with data experts. Unlike previous reviews that primarily focus on AI in clinical practice or limited geographical contexts, this work offers a comparative perspective across regions and educational levels, with attention to policy alignment, equity, and competency development.

This review will also explore strategies, institutional efforts that are aimed at promoting AI and ensuring its competencies within training programs for healthcare practitioners. The review will also evaluate the impacts of these interventions on how prepared the workforce is and how resilient it is. The appraisal will guide policy making, development of curriculum and all relevant partnerships for a sustained integration of AI into public health.

## 2 AI integration into public health curricula: a global overview

The integration of artificial intelligence (AI) into public health and allied educational programs represents a significant global trend, characterized by distinct regional advancements, benefits, and inherent challenges. In North America, the discourse surrounding AI in education extends specifically to medical and public health curricula, emphasizing a thorough understanding of prerequisite pathways and foundational exposure to AI concepts (30). While AI is comprehensively addressed within computer science and engineering disciplines, a critical need exists for its systematic integration into undergraduate medical education (31, 32). Current initiatives aim to bridge the existing knowledge gap by developing structured AI curriculum frameworks specifically designed for medical education (31). These frameworks are essential for guiding the instruction of future physicians, ensuring they acquire the fundamental knowledge and skills necessary for the effective integration of AI into clinical practice. This includes investigations into AI exposure within Canadian undergraduate medical education programs (33).

European countries are also actively exploring the incorporation of AI into public health education, recognizing its substantial potential to enhance public health functions. Discussions at prominent events, such as the European Public Health Conference, emphasize ongoing efforts to navigate the rapidly evolving AI landscape, overcome implementation barriers, and facilitate AI’s transformative capabilities within European public health systems (34). Studies involving medical students in countries like Germany, Austria, and Switzerland have probed their perceptions of AI in medicine and the imperative of integrating AI ethics into medical education (35). These investigations highlight the necessity of comprehensive education to equip future practitioners for the adept utilization of AI tools while concurrently addressing associated ethical complexities (35). Furthermore, insights from Romanian academics regarding the implementation of AI in higher education illuminate various advantages and disadvantages, providing valuable perspectives on the academic reception and challenges of AI integration within a European context (36).

Sub-Saharan Africa is experiencing a growing impetus for AI integration into public health education and workforce development, despite encountering unique regional challenges. There is a recognized urgency to identify and implement relevant practices that foster AI readiness across the region, given its comparatively disadvantaged position in leveraging AI for economic growth globally (37). Specific initiatives aimed at AI skills capacity-building and workforce development include the Africa AI Accelerator in Ghana and the Harambee Youth Employment Accelerator in South Africa (38). The application of deep learning and machine learning models is also being explored to enhance healthcare across sub-Saharan Africa, unveiling emerging opportunities, prevailing trends, and potential implications (39). Nonetheless, a notable scarcity of published literature exists concerning AI-based applications specifically deployed to improve public health education and healthcare within the region (39). Comprehensive reviews are also examining efforts to integrate AI into health informatics to bolster public health in Africa (40). Digital technology, including AI, is set to play a pivotal role in achieving sustainable human development across the continent (41). Illustrative cases, such as the analysis of AI program implementation in Ethiopian STEM schools, highlights the importance of establishing foundational AI knowledge (42). Extensive reports further detail the current state of AI capacity throughout Sub-Saharan Africa (43). Moreover, the broader context of public health doctoral education in Africa accentuates the need for augmented capacity within the public health workforce, rendering AI integration an indispensable component for advancing research and practical applications (44).

Across the Asian continent, AI is profoundly reshaping healthcare, including the nursing profession, by presenting opportunities to elevate patient care and optimize outcomes (45). Countries like Singapore, South Korea, Japan, and China exemplify the successful integration of AI-powered technologies, encompassing chatbots, virtual assistants, data mining, and automated risk assessment systems (45). This transformative impact extends to nursing education, with a strong emphasis on personalized learning, adaptive instructional approaches, and AI-enhanced simulation tools (45). While specific data for Southeast Asia are often subsumed within broader Asian trends, documented applications, and ongoing discussions about AI in medical education in countries such as Pakistan (33) offer critical insights into the challenges associated with integrating AI into medical curricula. Similarly, multi-country surveys in the Middle East, including Jordan, Saudi Arabia, the United Arab Emirates, and Egypt, provide general insights into health profession students’ knowledge and attitudes toward AI, as well as perceived challenges in its integration (46). In Japan, studies have also investigated the challenges and opportunities presented by AI in inclusive education (47). The application of AI to public health education remains a key area of focus across the continent (48).

The integration of artificial intelligence (AI) into public health is a dynamic and rapidly evolving domain that calls for innovative approaches to workforce development and skill building. To meet this need, we are seeing the emergence of specialized bootcamps and strong partnerships between universities and industry, all designed to equip professionals and students with the essential AI skills. Focused bootcamps are proving to be a quick and effective way to bring healthcare professionals and educators up to speed on AI. These intensive programs are structured to offer fundamental AI concepts, explore their practical uses in healthcare, and weave AI tools into existing educational frameworks. For instance, an “Artificial Intelligence Bootcamp” was created to educate faculty, aiming to prepare nursing educators with crucial AI knowledge and hands-on tools for curriculum integration (49). This includes using AI-powered simulations to train future nurses for a healthcare landscape increasingly shaped by AI (49). Such initiatives highlight the importance of ongoing professional development in AI for those working in public health, both in practice and in academia (50). Given how rapidly AI is transforming healthcare, nursing education absolutely needs to keep pace, and these bootcamps are a smart way to bridge that knowledge gap among faculty (49).

Collaborations between universities and industry are vital for building AI capabilities and making sure that educational programs truly reflect real-world applications and demands. These partnerships, often framed within models like the Triple Helix, emphasize the give-and-take relationships among academic institutions, the business world, and government in translating knowledge into innovation (51). Such joint efforts can lead to the creation of bespoke AI skills development programs and contribute significantly to workforce readiness (38). Numerous examples demonstrate the positive influence of these partnerships, especially in areas like Sub-Saharan Africa, where there’s a clear understanding of the need to leverage AI for economic growth and sustainable development (37, 38). For instance, initiatives like the “Africa AI Accelerator” showcase how a business accelerator model can be applied to cultivate AI skills (38).

These collaborations help seamlessly integrate AI into health informatics, leading to better public health outcomes by addressing existing challenges and exploring new opportunities through deep learning and machine learning models (40, 41). Academics also share their views on the pros and cons of bringing AI into higher education, further highlighting the importance of carefully considered and strategic university-industry engagements (36). These partnerships also extend to curriculum development, ensuring that public health education effectively incorporates AI topics and concepts. Comprehensive reviews highlight the necessity of structured AI curriculum frameworks for medical students, residents, and practicing physicians to ensure that AI is successfully integrated into clinical practice (31). Moreover, a multi-country survey explored health profession students’ knowledge, attitudes, and perceived hurdles related to AI integration in their education, signaling a global demand for these types of partnerships (46).

The integration of artificial intelligence (AI) competencies into educational systems, particularly within public health, exhibits varying degrees of standardization and accreditation. A significant challenge identified in the current landscape is the observed lack of structured AI curriculum frameworks, leading to inconsistencies in how AI topics are taught and learned across different programs and institutions (31, 46). A comprehensive scoping review on AI curriculum frameworks for medical students, residents, and practicing physicians highlighted that, among the reviewed educational programs, there was an absence of a guiding theory, pedagogy, or overarching framework (31). This suggests a notable gap in standardized approaches to AI education within medical training, which has direct implications for public health professionals who often share foundational medical knowledge. The study emphasized the critical need for a structured AI curriculum to ensure that future physicians possess the necessary foundational knowledge and skills for AI integration into clinical practice (31).

Furthermore, a multi-country survey exploring the perspectives of health profession students on AI integration in their education revealed perceived challenges, indirectly pointing to a lack of uniform guidelines or accreditation (46). These challenges can stem from a variety of factors, including differing levels of technological infrastructure and varying institutional priorities across regions, such as those in Pakistan and other resource-limited settings (29, 33). While some initiatives, like specialized AI bootcamps for faculty, aim to equip educators with essential AI knowledge and tools for curriculum integration, these efforts often represent localized or institutional-specific standards rather than broader, externally accredited competencies (49). The comparison of AI curricula, even within regions like North America, can reveal diverse prerequisite pathways and content emphasis, further illustrating the varied degrees of standardization (30).

Ultimately, the current educational landscape for AI competencies in public health is characterized by a fragmented approach. While there is a growing recognition of the need for AI integration, comprehensive and universally adopted frameworks for standardization and accreditation of these competencies remain an evolving area, demanding continued development and collaboration among educational institutions, professional bodies, and industry stakeholders.

## 3 Competency development: shifting skill sets in the public health workforce

The COVID-19 pandemic accelerated the evolution of public health competencies, pushing data science, digital literacy, algorithmic ethics, and AI governance from peripheral topics to core expectations of practice (52–54). Prior to 2020, many public health frameworks limited attention to these domains; however, post-pandemic revisions by accreditating bodies such as the Council on Education for Public Health (CEPH) and the Association of Schools and Programs of Public Health (ASPPH) now emphasize explicit learning outcomes in data analytics, informatics, and technology leadership (55, 56). In 2021, U. S. core public health competencies were updated to emphasize data analytics and technology skills (57). Globally, the World Health Organization’s digital health strategy 2020 to 2025 called for strengthening workforce digital skills, including offering training on AI ethics to health professionals (58–60). Recent evaluations of workforce readiness have consistently identified major deficits in data literacy, data governance, and systems thinking, revealing a disconnect between academic preparations and real-world practice needs (52, 61). According to NACCHO’s 2024 Public Health Informatics Profile, only 5 percent of local health departments currently use artificial intelligence, and fully 84 percent have no plans to adopt it in the coming year (60). This limited uptake is compounded by a workforce in which many staff lack formal data-science education and have little access to ongoing training on emerging technologies (60). These gaps point to an urgent need to modernize public-health training systems and to align academic curricula and in-service training with the practical competencies required to recruit, develop, and retain a technology-enabled public-health workforce. Systematic reviews likewise show that digital competencies now cut across all traditional public health competencies, leading reviewers to propose a new meta domain for specialized digital public health competencies and roles (54, 62).

A 2025 study on workforce development similarly found significant gaps in capacity and training, particularly around data science, and called for integrating data science and leadership content into public health training programs (52). Algorithmic ethics and AI governance have also emerged as essential domains for professional development, aimed at ensuring the responsible use of AI tools in public health practice. Experts are calling for dedicated training to address algorithmic bias, data privacy, and AI ethics in public health, warning of risks if these skills are lacking (29). There is not yet a globally agreed upon framework for digital-era public health competencies. However, In January 2023, the World Health Organization convened a Digital Health Competency Framework Committee to draft a global standard that spells out the digital skills required of policymakers, managers, and frontline health professionals—a move that signals formal integration of digital expertise into the expected competencies of the modern public health workforce (63–65).

Schools of public health have been pressed to modernize curricula to produce graduates with these emerging skills. Many programs launched curriculum reforms and new courses integrating public health with data science, informatics, and technology. For instance, Arizona State University established a School of Technology for Public Health that combines public health training with human-centered technology, systems engineering, and design, demonstrating a transdisciplinary approach (66). Academic and professional bodies have also updated their expectations. The WHO-ASPHER Competency Framework for the Public Health Workforce in Europe explicitly includes informatics and digital transformation skills, indicating a recognition of digital skills as essential competencies (65). In the United States, accreditation guidance has encouraged integrating informatics and data analytics into MPH training in line with revised core competencies (65). A 2020 review of educational frameworks identified at least 28 distinct digital health competency areas, ranging from basic IT literacy and health information management to digital communication and data privacy understanding of ethical, legal, and regulatory obligations, and safeguarding data privacy and security (65). These competencies form the foundation of a digitally competent public health workforce.

Accreditation guidance reinforced these shifts. CEPH in the United States now specifies competency in data analysis and informatics for MPH graduates (55), while ASPPH coordinates an inter professional initiative to update core competencies around digital transformation (56). In Europe, the WHO–ASPHER framework places information, communication and digital health alongside surveillance and policy as essential public health functions (65). Despite these efforts, adaptation remains uneven. Barriers such as limited faculty expertise, curriculum overcrowding, and uncertainty about which emerging topics to prioritize have slowed comprehensive curriculum overhaul (65). Nonetheless, momentum is building as the pandemic’s lessons sink in. Educators are increasingly embedding digital competencies across existing public health courses, including epidemiology, biostatistics, and health promotion, rather than confining them to standalone modules (65). For example, some programs integrate data visualization or big data case studies into epidemiology and biostatistics courses and incorporate social media and e-health content into health promotion training (65, 67). This mainstreaming approach aims to ensure every graduate attains baseline proficiency in digital tools.

For the current workforce, 2020 to 2025 saw an explosion of online continuing professional development. Asynchronous virtual courses and hybrid workshops delivered by CDC, WHO and national agencies reached tens of thousands of practitioners and produced significant gains in knowledge, confidence, and intent to apply new skills (67, 68). Another trend is the rise of micro-credentials and digital badges as flexible learning options. These are short, competency-based courses focused on specific skills, often culminating in a certification or badge. Universities have introduced micro-credential programs for public health practitioners, offering digital badges upon completion (69). Early adopters report that badges motivate participation and provide portable proof of competence, although concerns remain about standardization and employer recognition (70). Professional associations expanded their training offerings as well. APHA provides an extensive catalog of online courses and webinars for continuing education credit, covering contemporary topics like data visualization, big data ethics, and digital contact tracing (71). Many CPD initiatives also emphasize change management and leadership, underscoring that technology adoption must be accompanied by cultural and ethical competencies (56). Initial results are encouraging, but challenges remain. Initial evaluations are promising, but barriers—such as time constraints, limited internet access, and lack of funding—persist, emphasizing the need for stronger institutional support (72). Cross-sector partnerships are helping agencies deliver high-quality training at scale despite resource constraints (61). Continued investment in workforce development through accessible online learning, micro-credentials, and supportive policies is viewed as crucial to sustaining an agile and digitally competent public health workforce.

## 4 Benefits and early impacts of AI education in public health

Educational programs introducing artificial intelligence (AI) into public health include standard academic curricula as well as brief training sessions and self-study modules, designed to equip public health professionals with both technical and applied skills necessary to engage with AI systems effectively (73). These educational initiatives support students in developing technical skills as well as creating teams across disciplines while teaching ethical decision-making for public health goal achievement (26, 73). AI technologies, especially machine learning, detect hidden information in large datasets which results in earlier detection and more detailed prediction capabilities (74, 75). Such tools become effective only when users demonstrate their ability to read and execute predictions effectively. Public health officials trained in AI are better equipped to avoid misinterpreting model outputs or relying solely on automated systems, thereby improving response time and decision quality (76, 77).

The early COVID-19 pandemic demonstrated that areas equipped with AI and data science training capabilities used forecasting tools to speed up public health intervention delivery (77, 78). The surveillance of diseases gets transformed through AI because it unites and evaluates data from electronic health records alongside mobility information and social media content and environmental sensor data (79). Nonetheless, these AI-driven surveillance systems rely heavily on skilled public health professionals to validate outputs, manage operations, and interpret complex signals (79, 80). The implementation of AI-trained personnel leads to improved surveillance systems which increase sensitivity and decrease false positive results. Also, the implementation of algorithms for detecting abnormal hospital symptoms requires human assessment to eliminate incorrect interpretations and prevent public panic (81, 82). Professionals who have AI training demonstrate better ability to assess data origin while understanding its constraints which enables them to make decisions based on evidence (83, 84).

The incorporation of AI training into public health curricula in African and Asian countries has led practitioners to develop greater expertise in working with complex data systems and deliver interventions that match specific community requirements (85, 86). These positive developments encounter several ongoing difficulties. AI education remains unevenly distributed, often concentrated in high-income countries and elite institutions, which contributes to widening global health disparities (87). AI development threatens to intensify worldwide health disparities because developing regions could become left out from the digital revolution (87, 88). The implementation of AI systems in public health settings faces three major ethical issues which consist of algorithmic bias alongside data privacy concerns and explainability challenges (77, 89).

While AI presents transformative potential in public health, it also carries significant risks that require deeper consideration. One key concern is algorithmic bias, where AI systems trained on non-representative data can perpetuate or exacerbate existing health disparities (77, 90, 91). Additionally, the lack of transparency in AI decision-making—often referred to as the “black box” problem—can hinder accountability and reduce trust among healthcare professionals and the public (89, 92). Privacy breaches are also a major ethical concern, particularly as AI tools require large volumes of sensitive health data, posing threats to patient confidentiality if data governance frameworks are inadequate (93, 94). In resource-limited settings, premature deployment of AI without proper infrastructure or skilled oversight may not only lead to inaccurate outputs but also divert limited resources from more urgent health needs (95–97). As such, AI education programs must not only provide technical training but also engage learners in critical discussions around ethics, governance, and accountability. A workforce that understands AI functionality together with its appropriate utilization needs to be developed because this will help build trust and inclusion for upcoming public health initiatives (98).

Closer collaboration between public health professionals and data scientists is also emerging, driven in part by AI education. These partnerships are fostering new interdisciplinary roles that bridge technical and public health domains (54, 99). The rapid growth of digital health data has united these two fields into one combined discipline as health policies and health challenge solutions now heavily rely on big data and AI (100–102). What’s driving this change? A big part of it is education. Education plays a key role in this transformation, with many institutions incorporating instruction in machine learning, natural language processing, and predictive analytics into public health programs (54). These educational initiatives enable public health staff to collaborate better with data scientists which leads to joint work opportunities and creative solutions (103). The main advantage emerging from this transition involves enhanced communication capabilities (104). The joint knowledge base enables public health specialists to present their needs more precisely and data scientists to grasp the actual health issues they aim to resolve. The ongoing dialogue between these fields has transformed isolated partnerships into enduring partnerships that allow both parties to work together (104, 105). Artificial intelligence tools now serve three essential functions such as disease outbreak prediction, environmental hazard detection and high-risk health profile identification (106). Health departments leverage AI effectively and ethically through professionals who serve as health data translators and AI implementation specialists and public health ethicists (107, 108).

Despite progress, access to AI training remains limited in resource-poor regions. Moreover, AI adoption is hampered by weak integration between technological tools and day-to-day public health operations, along with ethical challenges around data use and algorithmic bias. Addressing these issues requires broader inclusion of voices from marginalized communities in the development and oversight of AI systems (109, 110). Encouragingly, as more public health professionals acquire AI competencies, they are increasingly influencing both the implementation and design of AI solutions (54).

Evidence also suggests that technology-forward health systems with AI-trained leadership are more prepared and adaptable during crises. The preparation of health leaders for digital transitions requires specialized programs which focus on developing managerial competencies (80). Better system adaptability emerges from leadership readiness. In a sample of thirty-three health systems, a faster adoption of telehealth together with AI-driven diagnostics and remote monitoring was made possible because they were leaders who demonstrated both dedication and digital competence (111). To support broader workforce adaptability, digital training must be embedded into both initial education and continuing professional development strategies (112). The lack of digital tool education in Africa creates unequal distribution of innovation benefits because of insufficient foundational digital training (113). Digital competencies become stronger when workers engage in simulation-based learning and team up with experts from different fields. Professional skills from various fields have received substantial advancement through interdisciplinary conferences that focus on explainable AI development (114).

Public health surveillance together with decision support demonstrates the most significant transformation in digital implementation. AI-enhanced surveillance tools during COVID-19 needed trained users to interpret and respond to and manage the data outputs (115). Similar applications exist in healthcare practices at large as healthcare data science requires continuous involvement of healthcare staff. Health professionals need training to utilize these technologies effectively within their specific healthcare context for outbreak forecasting and individualized patient interventions (116).

Technology-trained workforces have shown greater resilience during emergencies, particularly in underserved and rural areas where digital enablement has enabled flexible care delivery models (117). The COVID-19 pandemic demonstrated that healthcare teams with digital literacy could shift care approaches and sustain service operations when under duress. This agility extends to specialty care. The successful implementation of tele-dermatology in the U.S. Veterans Health Administration required both technological infrastructure and staff training to deliver the care model (118). These advancements create many benefits yet ethical concerns together with equity issues continue to exist. Health leadership must provide universal access to digital transformation benefits for all workforce members throughout low- and middle-income areas (119). The implementation of AI systems creates safety risks for patients when ethical safeguards are not in place because bias can persist in their operations. Technical training should be paired with ethical education for all professionals. Public health professionals need education that includes training them to detect algorithmic bias and protect patient confidentiality and promote AI system transparency (92, 98).

## 5 Persistent challenges and gaps

AI has demonstrated notable benefits in predicting epidemiological patterns and population needs (120). However, its implementation across developing and underdeveloped countries is hindered by a combination of infrastructural, educational, political, and ethical barriers. One of the most pressing challenges is infrastructural deficit and digital divide. Many low- and middle-income countries (LMICs) struggle with inadequate technological infrastructure, including unreliable internet connectivity, limited access to high-performance computing systems, and sociocultural barriers that impede technology use (95). The financial burden of buying, installing, and maintaining these devices and application are equally expensive and requires constant funding which poses as a challenge to adopting AI models in health education (96). According to research done by statistica in Uganda (121, 122), only 7.48 million in 2024 have access to internet with estimated internet penetrance of 14.9% which shows an enomous set back in use of AI which requires strong internet connection.

In addition to infrastructural limitations, insufficient specialist skill sets and low general digital literacy among healthcare professionals present significant obstacles. A study in Zimbabwe, for instance, revealed suboptimal ICT skills among medical doctors, which was linked to the low adoption of e-health systems (123). As a result of this inadequate skill, integrating AI into health education proves to be a challenge because the healthcare professional is expected to be knowledgeable in using digital skill to promote good health at individual and population level (124). Additionally, many rural populations lack smart devices, which further contributes to the challenge in LMICs (97). Despite limited knowledge by healthcare workers, they have shown a positive attitude towards accepting AI technology (125). This therefore shows that with the right training and support, these challenges can be addressed.

Political will and government commitment also play pivotal roles in the successful integration of AI into public health education. Investment in AI requires developers’ confidence and trust in its application and an enabling economy and environment. However, a lack of government commitment to creating enabling regulations and policies in promoting the adoption of health digitalization and AI incorporation stands to threaten the use of AI in health education (95). However, some LMICs have started implementing e-health but have limited policies to ensure data collection one that provides the framework of AI models specific for each country for population-tailored health education (41).

Ethical concerns are another persistent barrier, particularly around data privacy, algorithmic bias, and transparency. The functionality of AI in public health education requires gathering of large personal health data from patients which poses huge ethical concerns as it has a potential of breaching patient confidentiality, privacy, and security (93). Balancing data access with individual privacy rights is essential, and robust policy frameworks are needed to manage this tension. UNESCO’s ‘Guidance for the use of Generative AI in education and research’ emphasizes the importance of human-centric technology and national policy alignment to mitigate risks and ensure ethical AI use (126). Algorithmic bias remains a major concern, especially when datasets used to train AI systems lack cultural and demographic diversity. Such biases can lead to inaccurate or discriminatory outcomes, which may compromise patient safety and public trust in health systems. Studies have documented cases where AI algorithms showed bias against Black patients, highlighting the urgency of culturally sensitive data collection and model development (90, 91). Cross-cultural approaches to AI development are necessary to ensure fairness, accuracy, and inclusivity in educational and health applications (94).

Institutional resistance and slow curricular reforms further complicate efforts to embed AI into public health education. Barriers such as limited awareness, lack of trained experts, and disinterest from decision-makers hinder curriculum innovation (127). Traditionally, public health education is one of the key courses in public health taught in medical school which ensures outlined guidance for patient education. However, the growing demand for AI integration has sparked concerns over possible overreliance on technology and potential declines in critical thinking among students (128). As a result, striking a balance between adopting innovative AI tools and preserving core medical competencies remains crucial for sustaining the quality and integrity of public health training.

## 6 Recommendations and future directions

Since the COVID-19 pandemic, public health has progressively evolved globally, and the use of AI in academia has been discussed as crucial (129). In the past five years, there has been significant progress ranging from AI-driven surveillance and management of chronic diseases to the increasing incorporation of data science into academic curricula. Nonetheless, persistent challenges such as educational quality disparities and conceptual abstraction remain obstacles to equitable adoption (26). In response to these complexities, strategic, context-sensitive approaches are required to guide the global integration of AI into public health education.

A context-sensitive global framework for AI integration into curricula is needed to balance consistency with flexibility. Globally, the incorporation of AI into the creation of public health curricula provides a dynamic yet fixed educational model (26). To effectively achieve this, we proposed a tiered model for curriculum development which would be adaptive and sustainable globally. This tiered model includes the core framework, modular component, and Implementation scaffold. At its foundation, a core framework would encompass essential components, including basic mathematics, statistics, and introductory content on AI and machine learning (ML), ensuring that every learner gains fundamental literacy in AI concepts regardless of region (48). The management of highly sensitive and personal patient records has raised data privacy concerns; strict privacy measures and rigid encryption systems should be particularly included at this stage to avoid any data breaches (29). There is also the need to include the potential for bias in AI algorithms, especially racial bias, underscoring the need for risk awareness and ethical AI utilization (130). Complementing the core foundation, modular elements would provide adaptability to national priorities and available resources. The use of mobile and web-based AI platforms could be tailored to resource-limited countries (131) while more complex AI-centered programs like advanced analytics (132) and real-time surveillance (133) in resource-rich countries. Various AI specializations should be included as a component based on the nation’s needs and resources such as AI in epidemiology and, AI and environmental health surveillance. Furthermore, the use of AI should be targeted to public health concerns endemic to the region of concern such as Malaria and Dengue in Asia and Africa (134, 135) and the Opioid crises in the USA (136).

Implementation strategies must align with institutional capacities and national development goals. This involves practical application based on undergraduate and postgraduate levels of education. More complex AI implementations in public health are covered at this stage such as the practical implementation of tools like Python, and R and the use of AI in disease prevention, infection source control and health management (48). Given the rapid advancement of AI technologies, continuous curriculum evaluation is essential. Stakeholder feedback from students, educators, policymakers, and private-sector developers must guide these evaluations to maintain relevance and sustainability.

To advance this vision, strong partnerships between public institutions, academia, and the private sector are vital. Collaboration between policymakers, educational institutions and private AI developers has become a paramount aspect of advancing the future of AI in public health education (48). This multi-sectoral partnership would assist in the creation of a training module targeted at the alleviation of challenges ranging from inadequate skill, funding, and racial bias (29). Educational institutions have the role of leading cutting-edge AI-driven research, upskilling professionals through certification programs and workshops and promoting ethical literacy. Funding and resource allocation, establishing a regulatory framework for ethical AI use, capacity building and workforce planning through national training programs are the roles taken up by the policymakers. Private AI developers provide investment and funding, accessibility and scalability through localization and language support. The stakeholders can function synergistically to create a robust ecosystem for effective and sustainable integration of AI in public health education. Collaboration across these sectors would lead to the creation of curricula that are both technically current and contextually relevant to real-world needs, faster public health application, improved inclusivity across geographic and socioeconomic boundaries and the creation of a feedback loop which makes public health education sustainable and relevant.

Promoting equity-focused policy reforms is also essential to ensure that AI literacy does not deepen educational or technological inequalities. In alignment with the United Nations Sustainable Development Goal 4, which calls for inclusive and equitable education for all (137), AI integration must prioritize the inclusion of underserved and marginalized populations. This involves developing policies that attract private-sector investment in digital infrastructure within low- and middle-income countries (LMICs) by facilitating transparent public-private partnerships and establishing special economic zones to support digital expansion. Language localization and the creation of culturally appropriate teaching materials are necessary to address disparities rooted in cultural or linguistic barriers. Additional support mechanisms—such as scholarships, and remote learning platforms to students from LMICs and young girls would provide subsidized or free access to AI training programs. By ensuring broad access to AI-related education and tools, such policies can prevent the emergence of a global knowledge divide and promote equitable engagement in the digital transformation of public health.

## 7 Conclusion

Five years after the COVID-19 pandemic began, incorporating artificial intelligence (AI) into public health education is essential to developing a resilient and prepared health workforce. The progress made worldwide in integrating AI literacy into curricula, the cooperative frameworks influencing educational reforms, and the initial advantages shown in worker flexibility and data-driven public health decision-making are all highlighted in this paper. However, enduring issues, including inadequate infrastructure, unequal access, moral dilemmas, and inconsistent standards, continue to be urgent. Stronger public-private-academic partnerships, inclusive policies that give priority to marginalized groups, and context-sensitive frameworks are all necessary to ensure equitable, sustainable integration. Ultimately, a globally coordinated effort to integrate AI into public health education will not only enhance preparedness for future health crises but also ensure a digitally competent and ethically grounded health workforce capable of leveraging AI for population health advancement.
